# Low-Power Consumption IGZO Memristor-Based Gas Sensor Embedded in an Internet of Things Monitoring System for Isopropanol Alcohol Gas

**DOI:** 10.3390/mi15010077

**Published:** 2023-12-29

**Authors:** Myoungsu Chae, Doowon Lee, Hee-Dong Kim

**Affiliations:** 1 Department of Semiconductor Systems Engineering, Convergence Engineering for Intelligent Drone, Institute of Semiconductor and System IC, Sejong University, 209, Neungdong-ro, Gwangjin-gu, Seoul 05006, Republic of Korea; 2 IHP GmbH—Leibniz Institute for Innovative Microelectronics, Im Technologiepark 25, 15236 Frankfurt (Oder), Germany

**Keywords:** isopropanol alcohol gas, gas sensor, Internet of Things, monitoring, memristor

## Abstract

Low-power-consumption gas sensors are crucial for diverse applications, including environmental monitoring and portable Internet of Things (IoT) systems. However, the desorption and adsorption characteristics of conventional metal oxide-based gas sensors require supplementary equipment, such as heaters, which is not optimal for low-power IoT monitoring systems. Memristor-based sensors (gasistors) have been investigated as innovative gas sensors owing to their advantages, including high response, low power consumption, and room-temperature (RT) operation. Based on IGZO, the proposed isopropanol alcohol (IPA) gas sensor demonstrates a detection speed of 105 s and a high response of 55.15 for 50 ppm of IPA gas at RT. Moreover, rapid recovery to the initial state was achievable in 50 μs using pulsed voltage and without gas purging. Finally, a low-power circuit module was integrated for wireless signal transmission and processing to ensure IoT compatibility. The stability of sensing results from gasistors based on IGZO has been demonstrated, even when integrated into IoT systems. This enables energy-efficient gas analysis and real-time monitoring at ~0.34 mW, supporting recovery via pulse bias. This research offers practical insights into IoT gas detection, presenting a wireless sensing system for sensitive, low-powered sensors.

## 1. Introduction

While the world is presently experiencing a catastrophic energy crisis, the ongoing advancement of the Internet of Things (IoT) necessitates the integration of an immense quantity of sensors, which consumes an enormous amount of energy [1,2,3,4,5]. In considering the vast quantity of sensors that must be integrated into such a network, there is an immediate demand for sensors with the following attributes: micro- or nanoscale dimensions, continuously improving sensitivity and detectivity, significantly reduced response times, and power consumption that is orders of magnitude lower than that of existing commercial devices. Conventional gas sensors are still energized by a voluminous and inflexible external power source, which not only results in the expansion of the system’s overall dimensions but also significantly compromises the device’s portability and comfort. To optimize power efficiency and ensure extended device life in gas monitoring technology, low-powered functionalities are anticipated to be integrated into the sensor [6,7,8]. Chen H. et al., for instance, reported that the energy-storage capability of FMCPIB-based devices enables them to function as photo capacitors to detect NO_2_ for an additional 1.7 h in the dark without requiring an external power supply [8]. According to Cho et al., the operational power of the gas sensor composed of ZnO nanowires was ~184 μW, indicating that it can be utilized in practical IoT devices [9].

Integrating IoT systems into industrial safety protocols to monitor volatile organic compounds (VOCs) to avert potential industrial catastrophes has received considerable attention. These cutting-edge devices can identify and measure the levels of diverse species, including ethanol, NO_2_, and CO, in both biological fluids and the atmosphere [10,11,12]. They operate within field management systems that are low-power and high-density. Among various types of gas sensors, gas sensors based on metal oxide semiconductors have attracted considerable attention for detecting VOCs due to their rapid response time and broad sensitivity to various target gases [13,14]. The miniaturization and low cost of manufacturing metal oxide semiconductor gas sensors enable the implementation of high-density arrays in systems. Furthermore, it has been reported that applying nanomaterial-based metal oxides enhances the properties of sensors [15,16]. According to the results of Le et al., porous CoFe_2_O_4_ nanorods exhibited a large surface area for the reaction, resulting in an increased reactivity to acetone at 350 °C [17]. These oxides are distinguished by their comparatively high mobility of field effects, safety features, low-leakage current, and room-temperature (RT) process availability. As a result of their compatibility, ease of synthesis, and capacity for low-temperature processing, metal oxides have attracted significant interest for implementation in IoT systems [18]. The emphasis is especially placed on amorphous indium gallium zinc oxide (a-IGZO) due to its potential as an active channel material. This is primarily due to the favorable attributes of its amorphous phase, including exceptional uniformity, long-term stability, and flexibility. Cho and N. G. demonstrated that, at an operating temperature of 300 °C, a gas sensor comprising a semiconductor a-IGZO tube network showed n-type gas-sensing properties and a 3.7-fold increased gas response in comparison to a planar a-IGZO thin film (R_Gas_/R_Air_ = 29.4) [19]. However, notwithstanding their numerous advantages, the critical high-temperature operation of these gas sensors based on metal oxides has restricted their use in ubiquitous gas monitoring systems. Ensuring high gas sensitivity and fostering surface redox reactions are contingent upon this temperature range. Additional devices, such as ultraviolet or heaters, are necessary to apply this energy, increasing the volume and power consumption. Consequently, further improvements are required to implement IoT monitoring techniques for low-power consumption and operating at RT.

Gasistors, memristor-based gas sensors that combine a gas sensor and a memristor, have been recently introduced. Characterized by their distinctive detecting mechanism that diverges from traditional gas sensor approaches that rely on variations in the width of the depletion region on a surface, gasistors have garnered significant interest owing to their low power consumption, high sensitivity, and rapid recovery time [20,21,22]. They can be a feasible resolution as they boast a fast response time at RT and a high sensitivity towards the target gas. It is widely acknowledged that a variation in the resistance of a gasistor can be attributed to the creation or rupture of vacancy paths, which consist of conducting filaments (CFs) and oxygen and nitrogen vacancies in the thin film [23,24]. By employing nano-sized CFs for gas-sensing, gasistors can identify a target gas with a minimal sensing current at RT and produce a robust response even for trace gases, thereby conserving energy. Chue et al. reported a memristive gas sensor of TiO_2_ nanorods at RT exhibiting rapid response and quick recovery time. [25]. Qiu et al. realized an extremely fast response and recovery speed of 1.2 s through an ultrasensitive gas sensor developed from a SnS/TiO_2_-based memristor [26]. Furthermore, in one of our prior investigations [27], we documented a rapidly recovering and detecting IGZO-based gasistor at RT, in addition to a velocity of 1 s/90 ns. However, IGZO-based gasistors for VOC gas monitoring systems have not yet been investigated, and their research on low-power monitoring systems for IoT is limited.

**Figure 1:**
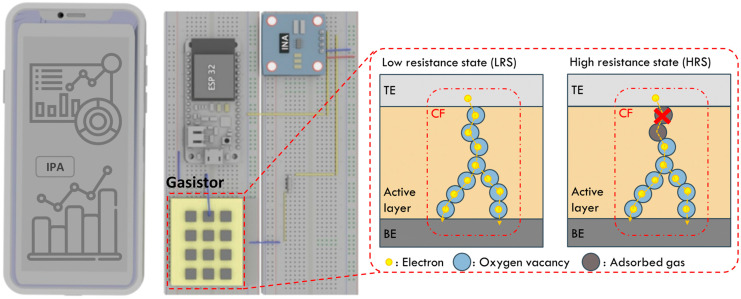
Conceptualization of an artificial IGZO-based gasistor embedded in an IoT gas monitoring system. The schematic illustration on the right shows the gasistor-based IPA gas monitoring system as well as the reaction and recovery mechanisms of the gasistor devices. The image on the left depicts a mobile device that employs an IoT to monitor IPA gas levels in the air in real time.

In this study, we examined isopropanol alcohol (IPA) gas, one of the VOCs in IGZO-based gasistors. First, the unit device’s sensitivity and response characteristics concerning IPA’s concentration were initially assessed. Applying a pulse voltage allows the gasistor to be restored to its initial state even after the CFs rupture in response to the gas. During this investigation, the gasistor was returned to its original condition by applying a pulse with a width of 50 μs and an amplitude of 1 V. Furthermore, an IGZO-based gasistor was incorporated into the system based on the evaluated properties, and the results of the measurements were observed remotely using a mobile device. Consequently, the IGZO-based gasistor embedded in the IoT system achieved accurate measurement of the applied concentration and restored the initial state by applying voltage from a mobile device, as shown in Figure 1. The potential of implementing real-time monitoring using gasistor-based gas monitoring systems with operating voltages as low as 0.34 mW suggests the challenge posed by the high-power consumption of conventional semiconductor-based gas sensors could be overcome.

## 2. Materials and Methods

First, to prepare the IGZO-based gasistor, the Si substrate was cleaned using acetone, methanol, and deionized water for 10 min, respectively. The Pt bottom electrode (BE), which was 100 nm thick, was deposited on the cleaned SiO_2_/Si substrate using an electron-beam evaporation system. A 100 nm IGZO was then deposited on the Pt/SiO_2_/Si substrate using an RF sputtering system. The IGZO deposition was performed at a 100 W sputtering power in 20 sccm of Ar ambient at a base pressure of 20 mTorr and a working pressure of 5 mTorr. The compositional ratio of the sputtering target was In_2_O_3_:Ga_2_O_3_:ZnO = 1:1:1 (VTEX) with 99.99% purity. Subsequently, the 8 nm Ti top electrode (TE) was deposited on IGZO/Pt/SiO_2_/Si substrates using an RF sputtering system. A Field Emission Scanning Electron Microscope (FE-SEM, SU8010, Hitachi, Tokyo, Japan) was used to obtain a cross-section image of the gasistor to validate that the device had been deposited as planned. In order to evaluate the material characteristics of the IGZO film, the deposited IGZO film was analyzed using an X-ray diffractometer (XRD, Empyrean, Panalytical, Malvern, UK). The IGZO thin film’s chemical states were analyzed utilizing X-ray photoelectron spectroscopy (XPS, NEXSA G2, Thermo Fisher, Waltham, MA, USA). 

To evaluate the resistive switching (RS) capacity, we measured the electrical characteristics of the IGZO-based gasistor using a Keithley 4200 Semiconductor Characterization System (SCS). To demonstrate the gas-sensing capability of the IGZO-based gasistor at RT, the current of the IGZO-based gasistor was monitored at a read voltage of 0.2 V while inducing IPA gas. The IGZO-based gasistor was placed on the ground plate inside the gas-sensing chamber and electrically connected to a Keithley 4200 SCS. In the gas-sensing test, a 30 cm^3^ aluminum chamber was used, which was isolated from the outside environment. The chamber was evacuated with air for 50 s before the injection of IPA gas. Next, air and IPA gas were introduced into the chamber to assess the response properties. The target and air gas circulated until there was no further current variation for 50 s, allowing the gasistor to react completely. The concentration of IPA gas was adjusted from 10 to 50 ppm. The object was returned to its original state by implementing a pulse bias. We optimized the pulse voltage and applied a pulse with a size of 1 V and a width of 50 μs. Each of these procedures was replicated for each gas concentration procedure. To examine the response characteristics of the concentration, we adjusted the air and target gas flow rates while maintaining a constant total flow rate of 500 sccm. The humidity was fixed and maintained at 36% relative humidity (RH).

The microcontroller (ESP 32) and current sensor (INA 219) were connected to a breadboard for monitoring and communication. IPA gas was detected within the chamber by the IGZO-based gasistor, which subsequently notified the mobile device of the current change. The mobile device determined the concentration of IPA gas in the chamber based on the real-time values received from the IGZO-based gasistor. The IoT module transmitted the current change value of the gasistor in the chamber to the mobile device every 2 s, allowing for real-time monitoring of IPA gas. The incoming data included current, voltage, power consumption, and concentration on the mobile device through the Blynk application.

## 3. Results and Discussion

**Figure 2:**
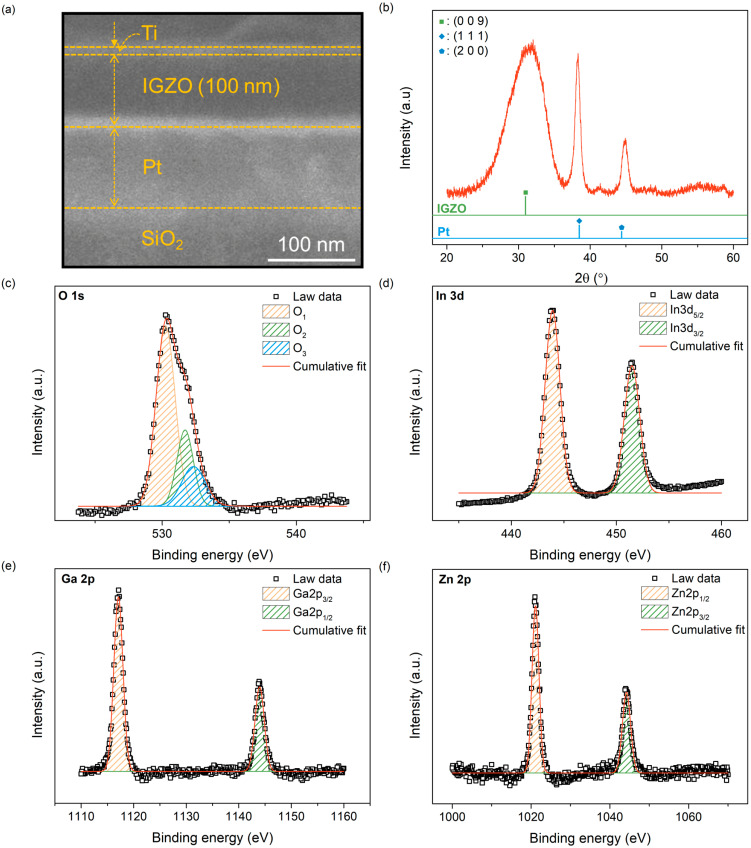
Material characteristics of IGZO-based gasistor. (**a**) Cross-section FE-SEM image with a scale bar of 100 nm. (**b**) XRD result of IGZO film. XPS spectra peaks of (**c**) O 1s, (**d**) In 3d, (**e**) Ga 2p, and (**f**) Zn 2p in IGZO film.

Before describing the resistive switching (RS) and gas-sensing characteristics of the IGZO-based gasistor, the material properties of IGZO were investigated. A cross-sectional FE-SEM image, shown in Figure 2a, was measured to evaluate the fabricated device structures, confirming each layer. Furthermore, to analyze the structural characteristics of the IGZO film, the deposited IGZO film was analyzed using an X-ray diffractometer (PANalytical, empyrean). As shown in Figure 2b, the IGZO/Pt sample shows diffraction peaks with 2θ values at around 31.46°, 38.30°, and 44.85°. A diffraction peak located at 31.46° (JCPDS 01-070-3626; [28]) shows the (009) IGZO film, which is consistent with other previous studies [29]. At 38.30° and 44.85°, the crystalline phases were identified as the (111) and (200) planes, respectively, of face-centered cubic bulk metallic counterparts [30]. The mean crystallite size of the (009) IGZO film was 1.47, calculated using Bragg’s law. To confirm the chemical composition of the IGZO, we investigated the XPS peaks to analyze changes in the binding energy of atomic orbitals, which can be related to changes in the chemical environment of the atoms. Figure 2c–f show that O, In, Ga, and Zn oxidation states are analyzed using their respective high-resolution spectra. As obtained through XPS analysis, the O 1s spectra of a-IGZO can be separated into three synthetic signals, as shown in Figure 2c. Our analysis showed that the binding energies for the three signals were 530.1, 531.7, and 532.3 eV. The binding energy peaks reveal fully oxidized oxygen (M-O) at 530.1 eV, oxygen vacancies at 531.7 eV, and oxygen in the hydroxide state (M-OH) at 532.3 eV, as indicated by the binding energy peaks [31,32]. Two peaks centered at binding energies of 444 eV and 451.5 eV, respectively, in the XPS data for the In 3d signal are shown in Figure 2d. These peaks are the In 3d5/2 and In 3d3/2 doublets, with an orbital splitting of 6.5 eV. The peaks of Ga 2p and Zn 2p was found to be around 26.9 and 23 eV, respectively, which is consistent with previous studies [33].

**Figure 3:**
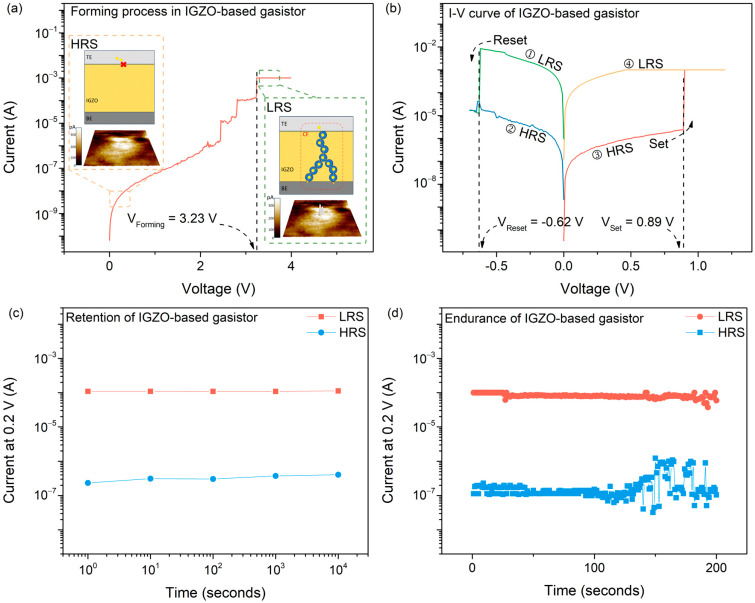
Schematic illustration and RS characteristics of an IGZO-based gasistor. (**a**) The forming process of the IGZO-based gasistor, with the enlarged figure showing the atomic structure changes in the IGZO-based gasistor before and after the CF forming process measured by C-AFM; (**b**) I–V curve; (**c**) retention; and (**d**) endurance characteristics of the IGZO-based gasistor.

Subsequently, the RS of the IGZO-based gasistor was accessed by measuring the current–voltage (I-V) curve characteristics of a DC voltage sweep with a Keithley 4200 SCS. The morphology of the CF was also examined using a conductive atomic force microscope (C-AFM). C-AFMs enable a more dependable evaluation of the evolutionary behavior of a single filament due to the rarity of multi-filament events (which are frequent at the device level) in such a tiny probing area [34]. According to the CF model, the resistance of the IGZO can be changed in the opposite direction from HRS to LRS by implementing a setup or reset procedure. In this study, the IGZO film was used as an insulator. An initial DC bias sweep was performed on the IGZO-based gas resistance between BE and TE to determine the optimal voltage for CF formation, as shown in Figure 3a. During this process, oxygen ions (O^2−^) in the IGZO film migrated towards TE while oxygen vacancies (V_o_^2+^) persisted. As the concentration of V_o_^2+^ exceeded a threshold, it underwent a self-reorganization process, resulting in the formation of CFs within the IGZO film, as shown in the magnified inset of Figure 3a. Next, we measured the I-V curve of the IGZO-based resistor under DC bias sweeps (0 V → −0.7 V → 0 V → +1.2 V), as shown in Figure 3b. A reset operation occurred when the resistance state of the resistor was changed from LRS to HRS due to a DC bias sweep being conducted from 0 V to −0.7 V. The sudden decrease in current during the reset procedure can be ascribed to the Joule heating-induced rupture of the CFs at −0.62 V. Conversely, following the re-sweeping of a DC bias from −0.7 V to 1.2 V, the resistance state of the gasistor was changed to LRS, an operation known as the set process. The abrupt surge in current magnitude at 0.89 V can be attributed to the reformation of the CFs occurring within the IGZO film. As depicted in Figure 3c, the current at LRS and HRS was monitored for 10^4^ s at a read voltage (V_read_) of 0.2 V to determine whether the CFs were maintained in the ambient. Consequently, we noted that the CFs remained stable for 10^4^ s in the gasistor, suggesting that they remained unreactive with the surrounding gases before injecting the IPA gas. As shown in Figure 3d, the endurance test was performed at the V_read_ for 200 DC cycles per resistance state to further assess the gasistor’s dependability. The HRS exhibited variability following more than 100 cycles; this is an intrinsic characteristic of oxide-based memristors, which, according to the principles of atomic motion physics [35], are susceptible to state variability and inhomogeneity issues. Nonetheless, the large ratio between HRS and LRS prevents problems such as read errors stemming from this volatility [36]. As a result, a 100 nm thick IGZO-based gasistor shows stable operation without any failures during 200 cycles. 

**Figure 4:**
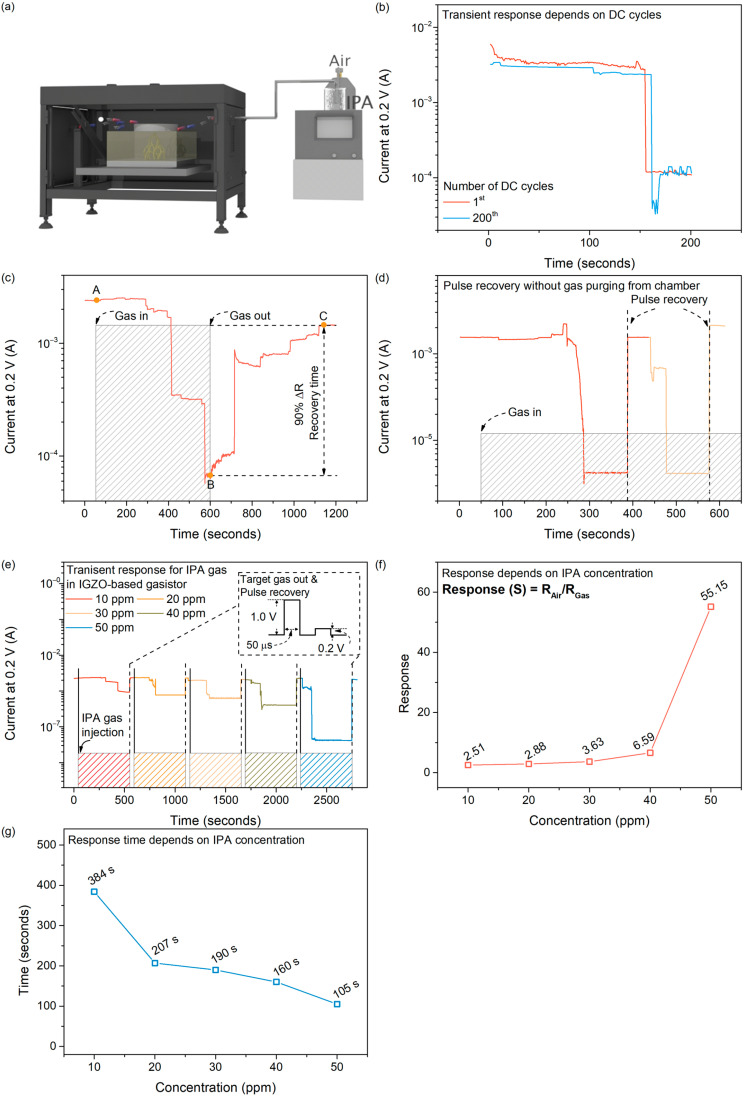
(**a**) Schematic structure of the gas-sensing environment. (**b**) A transient response based on the number of DC cycles to verify the stability of a gasistor. (**c**) Transient response testing to validate recovery time of IGZO-based gasistor. (**d**) Pulse recovery test without gas purging from chamber. (**e**) Transient response characteristics of IGZO-based gasistor for IPA gas depends on concentration. (**f**) Response and (**g**) response time depends on IPA gas concentrations in IGZO-based gasistor.

Next, the proposed gasistor’s transient response for IPA gas is monitored to study the gas-sensing characteristics, as shown in Figure 4a. Temperature and humidity substantially impact the gas-sensing abilities of semiconductor gas sensors, as is common knowledge [37,38]. On the other hand, prior research has demonstrated that gasistors exhibit relatively low sensitivity to changes in temperature and humidity [21]. Despite this immunity, humidity was maintained at 36% RH during gas sensing, and temperature was fixed to RT to exclude the effects of temperature and humidity. Then, to assess the stability, the sensing characteristics according to the number of RS operations were examined, as shown in Figure 4b. Thus, it was observed that the response time and current change in the device that initially formed CFs and reacted to 50 ppm gas and the device that broke and reformed CFs after conducting 200 DC cycles were nearly identical. We thus confirmed that reliability sensing can be achieved despite the carrying out of numerous operations. To validate the recovery time of the proposed gasistor, test sensing results are shown in Figure 4c. Following the injection of dry air gas for 50 s, IPA gas was injected with air gas at point A. Following a 500 s release of the target gas, IPA gas was purged at point B. The point required to recover 90% of the total resistance change was applied to determine the recovery times [39]. We observed that the IGZO-based gasistor, as proposed, returned to its original state approximately 543 s after the purging of IPA at point C. Nevertheless, previous work has demonstrated that CFs can be reformed via voltage application, thereby avoiding slow recovery times. Therefore, we applied a pulsed bias to restore it to its initial state to accelerate the process following the gas reaction. In addition, we examined whether a pulse voltage could restore the gasistor in the chamber to its initial state without purging. Figure 4d shows this with a continuous injection of 50 ppm of IPA gas after 50 s. Then, the initial state was reinstated 100 s after the reaction with the gas by applying a pulse voltage without gas purging. Consequently, it was validated that the initial condition could be reinstated by applying a pulse voltage, even in an environment containing gas. Furthermore, the subsequent reaction time decreased due to the absence of gas purging. This demonstrated that the formation of CFs in response to voltage application could restore the system to its initial state, even in an environment with continuous gas flow. Figure 4e shows the transient response to the IPA gas, where the gas concentration ranged from 10 to 50 ppm. The resistive state stability was observed for 50 s while air gas was introduced. Following the injection of IPA gas for 500 s, the IPA gas supply was discontinued, and a recovery pulse voltage was concurrently applied to reinstate the initial state. As a result, we observed trends where the current decreased from the initial state as the gas when the concentration of the IPA gas increased. When 10 ppm of IPA gas was introduced into the IGZO-based gasistor, IPA was adsorbed onto the surface of the IGZO film. The adsorbed IPA gas possessed a negative charge due to its property as a reducing gas [40,41,42]. Following adsorption with a negative charge, the IPA gas underwent a chemical reaction with V_o_^2+^, creating ruptured CFs. As a result, the reduction in current observed was ascribed to the existence of these fragmented CFs. The observed decrease in current with increasing IPA concentration can be attributed to the increase in the destructed CFs caused by the adsorbed IPA gas. To conduct a quantitative assessment of the response (S) characteristic, S = R_Gas_/R_Air_ was subsequently examined, where R_Gas_ and R_Air_ represent the resistances of the gasistor before and after IPA flow, respectively, during air injection, as shown in Figure 4f. The results demonstrated that the response increased with IPA concentration, reaching its maximum value of 55.15 at 50 ppm. The high response of pristine In_2_O_3_ to isopropanol could be attributed to the low C-C bonding energy of isopropanol (345 KJ·mol^−1^) compared to the (O-H) bond in methanol (458.8 kJ·mol^−1^) and the (C=O) bond in acetone (798.9 kJ·mol^−1^) [43,44]. The response time graphed against IPA concentration is shown in Figure 4g. With increasing gas concentrations, the device’s response time decreases. This could be attributed to the increased gas concentration, which causes a corresponding rise in the diffusion rate from the TE to the CFs. At a concentration of 10 ppm, this test required 384 s to detect the current change. At 50 ppm (the maximum concentration we assessed), the current change occurred in 105 s.

**Figure 5:**
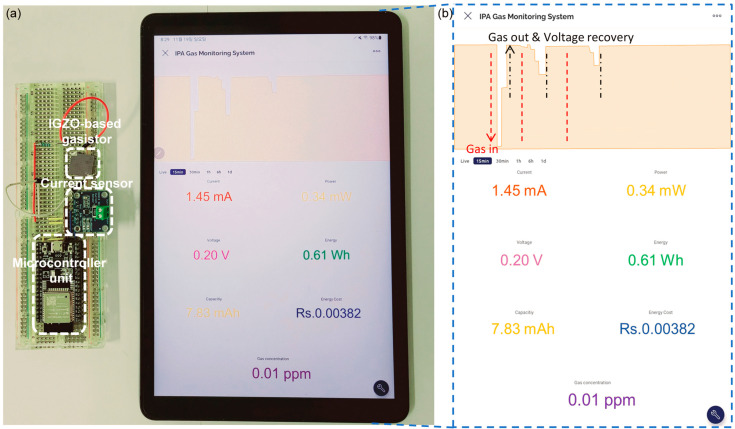
(**a**) Schematic representation of an IoT IPA gas monitoring system with an IGZO-based gasistor. (**b**) Real-time monitoring characteristics using an IoT system with an IGZO-based gasistor depend on IPA gas concentrations.

Finally, an IoT real-time monitoring system for IPA gas was integrated with an IGZO-based gasistor. This system incorporates an Arduino microprocessor, a current sensor, and an IGZO-based gasistor for IPA gas monitoring, as shown in Figure 5a. Figure 5b shows the effective monitoring of an unidentified reference gas through the utilization of the proposed system in conjunction with a mobile phone. This demonstrates that the IoT can measure IPA concentration in real time. In this context, we have effectively shown an IPA gas-sensing system for real-time monitoring and IoT systems. Users can obtain accurate concentration measurements via a smartphone application developed by the system. Table 1 shows a power consumption comparison of VOC gas sensors. Regarding power consumption, the evaluated monitoring system exhibits a negligible ~0.34 mW, which is notably low compared to established semiconductor gas sensors that rely on microheaters [45,46]. However, our proposed system’s power consumption is more significant than that of semiconductor gas sensors that utilize self-heating nanowires [47,48]. Nanostructures with a high surface-to-volume ratio are crucial for fabricating gas-sensing devices with exceptional performance [49,50]. In addition, nanostructures facilitate the efficient and rapid diffusion of gases through their network, consequently enhancing the surface area accessible for gas sensing. Nevertheless, the fabrication process for these gas sensors based on nanostructures or nanomaterials is exceedingly complicated, whereas the proposed gasistor retains the benefit of being simple to manufacture. Although gasistor-based systems are still in their infancy, it is possible to improve their power consumption by optimizing word lines and bits to reduce series resistance. Furthermore, gasistor can benefit from applying nanostructures, membranes, and nanomaterials typically used in semiconductor gas sensors, allowing for additional power consumption enhancements.

## 4. Conclusions

Gas sensors are indispensable constituents in many applications, encompassing environmental monitoring and portable IoT systems. However, achieving sustainable measurements and minimizing power consumption pose a significant challenge. Gasistors have been studied because of their beneficial attributes, including rapid response time and operation at RT. Through the integration of an IGZO-based gasistor into an IPA real-time gas monitoring system, we successfully demonstrated the sensor’s low power consumption. The proposed IPA sensor based on IGZO exhibited a notable response of 55.15 and a rapid detection speed of 105 s at RT. Furthermore, recovery to the initial state was possible in 50 μs even when pulsed voltage was applied in the presence of gas. To ensure compatibility with the IoT system, the proposed device incorporated a low-power circuit module designed to wirelessly transmit, modulate, and process signals. Thus, the integration of an IGZO-based gasistor into the proposed IoT system enabled gas analysis and real-time monitoring with a power consumption of less than ~0.34 mW. The primary purpose of this module with an IGZO-based gasistor is to facilitate the incorporation of remote and early warning systems in the event of a gas release while monitoring gas concentrations in the IoT system. This research paper contributes insights into gas detection by demonstrating a practical approach to constructing a wireless sensing system with a sensitive gas sensor that is both low-powered and energy-efficient.

## Figures and Tables

**Table 1:** Comparison of VOC gas sensors.

Target Gas	Sensing Material	Driven Source	Concentration (ppm)	Response (R_gas_/R_air_)	Response Time	Recovery Time	Power Consumption	Ref.
Benzene	SnO_2_@Au	415 °C	0.005	~4	~50 s	~50 s	8.9 mW	[46]
CH_4_	SiNWs/ TiO_2_	RT	120	1.5	75 s	191 s	-	[49]
Methanol	sulfonated RGO hydrogel	RT	0.005	1.40	-	-	20 nW	[50]
VOC	Fe_3_O_4_	300 °C	0.6	~1.2	6.1 s	10.7 s	92 mW	[51]
IPA	a-IGZO	RT	10	2.51	384 s	50 µs	0.34 mW	This work

## Data Availability

Data are contained within the article.
